# Young basketball players have better manual dexterity performance than sportsmen and non-sportsmen of the same age: a cross-sectional study

**DOI:** 10.1038/s41598-023-48335-7

**Published:** 2023-11-28

**Authors:** Alessandra Amato, Valerio Giustino, Antonino Patti, Patrizia Proia, Tatjana Trivic, Patrik Drid, Anja Obradovic, Marko Manojlovic, Maurizio Mondoni, Antonio Paoli, Antonino Bianco

**Affiliations:** 1 Department of Biomedical and Biotechnological Sciences, Section of Anatomy, Histology and Movement Science, School of Medicine, University of Catania, 95123 Catania, Italy; 2 Sport and Exercise Sciences Research Unit, Department of Psychology, Educational Science and Human Movement, University of Palermo, Via Giovanni Pascoli 6, 90144 Palermo, Italy; 3 Faculty of Sport and Physical Education, University of Novi Sad, 21000 Novi Sad, Serbia; 4 Department of Psychology, Catholic University of Milan, 20123 Milan, Italy; 5 Department of Biomedical Sciences, University of Padua, 35131 Padua, Italy

**Keywords:** Paediatrics, Public health, Quality of life, Movement disorders, Cognitive control, Perception, Motor control, Oculomotor system, Sensorimotor processing

## Abstract

Manual dexterity is a key skill in motor development. There are conflicting studies on the influence of sports practice on this skill and on which type of sport trains this ability the most in youth. Manual dexterity is usually assessed with expensive and time-consuming tools not easily available to facilities such as schools or sports clubs. The aim of this study was to assess differences in manual dexterity performance between young basketball players, sportsmen, and non-sportsmen. A further aim was to analyze whether the coin rotation task was a reliable tool for assessing manual dexterity. Based on the characteristics of the sport, we hypothesized that basketball players had better manual dexterity performances. Seventy-eight participants were included in the study and categorized into “basketball”, “sports”, and “non-sports” groups. Manual dexterity was assessed with the grooved pegboard, the coin rotation task, and the handgrip tests. The basketball group showed better performance in all tests. Significant differences were found between the basketball group and sports group and between the basketball group and non-sport group in the grooved pegboard (p < 0.05) and in the handgrip (p < 0.05) tests. Test–retest reliability of the coin rotation task scores was moderate in the basketball group (ICC_2,1_ 0.63–0.6). Basketball practice could positively influence manual dexterity. The coin rotation task showed an acceptable construct of validity.

## Introduction

Manual dexterity is the ability to manipulate objects through hand and finger coordination and is indicative of well-developed neuromotor function. It is the result of the functioning of different capacities such as cognitive function, muscle strength, and force control^1,2^. Fine hand skills are related to the different activities that people perform throughout their lives and they are fundamental for the development of self-efficacy and autonomy in the activities of daily living in young people. For example, the quality of children’s handwriting is highly dependent on manual dexterity and this can influence children’s participation in academic activities^3,4^.

There is now ample evidence of the beneficial effects of sports practice, especially in the early stages of life, on motor development and the maintenance of an adequate state of health^5–7^. However, the studies that associate physical activity or sports practice with the development of manual dexterity are conflicting in terms of results and, furthermore, it is not clear whether one sport can be more efficient than another. For example, Ziviani et al. investigated the association between daily physical activity and movement skills in children aged between 6 and 12 years, finding no significant correlation between manual dexterity tasks and daily physical activity^8^. George et al. investigated the effect of 6 weeks of physical activity, practiced with multimedia supports, in children aged between 6 and 12 years, finding an improvement in manual dexterity performance in males only but not in the entire sample which included males and females^9^. Kuloor showed no difference in eye and hand coordination (i.e., dexterity) between sportsperson and non-sportsperson^10^. Although these studies analyzed the influence of physical activity or sport on manual dexterity, practicing a specific sport may stimulate manual dexterity differently. The protocols of some study have demonstrated a significant effect on the development of manual dexterity using exercises with similar characteristics to a specific basketball training session of^11,12^. Basketball is a sport that requires eye-hand coordination, bimanual and visuomotor coordination, and manual dexterity to perform dribbling, passing, catching, throwing, shooting, and movements in dynamic and changing environments^13–15^. Basketball training involves manual dexterity and hand–eye coordination skills and determines a physiological adaptation of the brain nuclei involved in learning these skills. In fact, it has been demonstrated that the constant practice of basketball causes an increase in striatum and cerebellar volume in players which does not occur in inactive people^13,16^. Indeed, the cerebellum is involved in motor learning^16^ and the striatum plays a key role in motor learning, in the long-term memorization of well-learned movements, in the acquisition of manual and visuomotor skills, and in the bimanual coordination skill^17–21^.

Based on these physiological adaptations, it would be reasonable to hypothesize better manual dexterity in basketball players compared to inactive peers. Moreover, manual dexterity performance in young basketball players has not been studied and compared to that of those participating in other sports.

Hence, the first aim of this study was to investigate manual dexterity in young basketball players and to compare the performances to sportsmen and non-sportsmen of the same age. This could be useful for monitoring performances and manual dexterity development and possibly guiding parents in choosing the most appropriate sport for their children.

Furthermore, there are several neuromotor tests to measure manual dexterity with different efficacy according to age. Among them is the time it takes to complete the grooved pegboard test (GPT)^22^ or the most recent and simplest is the coin rotation task (CRT)^23^. The validity of this task has been studied in subjects with stroke, multiple sclerosis (MS), unilateral cerebral lesions^24^, and in children with specific learning disorders^23^. However, no studies have used this test to measure manual dexterity in young basketball players. Furthermore, the handgrip test (HGT) is considered an important indicator of upper extremity function^25^ and is often used as a diagnostic criterion for neurodegenerative diseases^26,27^.

For this reason, based on the training characteristics of basketball and through the use of these three tests (i.e., GPT, CRT, and HGT), the second aim of this study was to investigate the reliability of the CRT to establish a starting point for considering this test a cheaper and easier tool to measure manual dexterity performance in young active population of all contexts, even in schools or sports clubs.

## Methods

### Study design

For this cross-sectional study, we collected data from the recruited sample at one time point.

### Recruitment process

150 participants were assessed for eligibility between November 2022 and February 2023 in Sicily (Italy). In detail, we contacted by telephone the parents or guardians of: (a) 50 participants from the lists of 8–15 year category provided by the coaches of a basketball club in Palermo (Sicily, Italy); (b) 100 participants from the lists of 5 classes provided by the school principals of two Sicilian schools randomly chosen. Then, written information was sent.

Before starting the manual dexterity assessment, all participants filled out a questionnaire. The questionnaire was administered online via a link, and it was asked to indicate age, sport practiced, years of sporting activity (continuous), with which hand they write, and lateralization. From the responses, we categorized participants into the basketball group (BG), sports group (SG), and non-sports group (nSG). Specifically, participants included in the SG practiced soccer, volleyball, dance, gymnastics, karate, and kickboxing, but not basketball. This choice was made to include in the SG eye-hand and eye-foot coordination sports and sports with free-body exercises without tools. The inclusion and exclusion criteria for each group are reported in Table 1.

**Table 1:** The inclusion and exclusion criteria need to be included in the study for each group.

Inclusion and exclusion criteria for each group		
Basketball group	Sport group	No sport group
Inclusion criteria		
To play basketball for at least two years at least 2 times a week (minimum total 120')	To play sports (not basketball) for at least two years at least 2 times a week (minimum total 120')	No play sports for at least two years
Age between 8 and 15 years old	Age between 8 and 15 years old	Age between 8 and 15 years old
Exclusion criteria		
Neurological pathologies	Neurological pathologies	Neurological pathologies
Certified learning disorders	Certified learning disorders	Certified learning disorders
Limb injuries in the six months prior to the assessments	Limb injuries in the six months prior to the assessments	Limb injuries in the six months prior to the assessments
Cognitive impairments	Cognitive impairments	Cognitive impairments

### Assessments

Participants belonging to the BG were evaluated, in a single data collection session, in the sports facility where they usually train. The choice to recruit the BG in a single sports club was made to reduce bias related to any differences in the management of basketball training. The SG and nSG were evaluated in school classrooms.

The total duration of each evaluation was approximately 15/20 min during which the participants filled out the questionnaire and carried out the three manual dexterity tests (i.e., GPT, CRT, and HGT). The participants were familiarized with the tests by trying them once for each limb before data collection.

#### Handgrip test (HGT)

**Figure 1:**
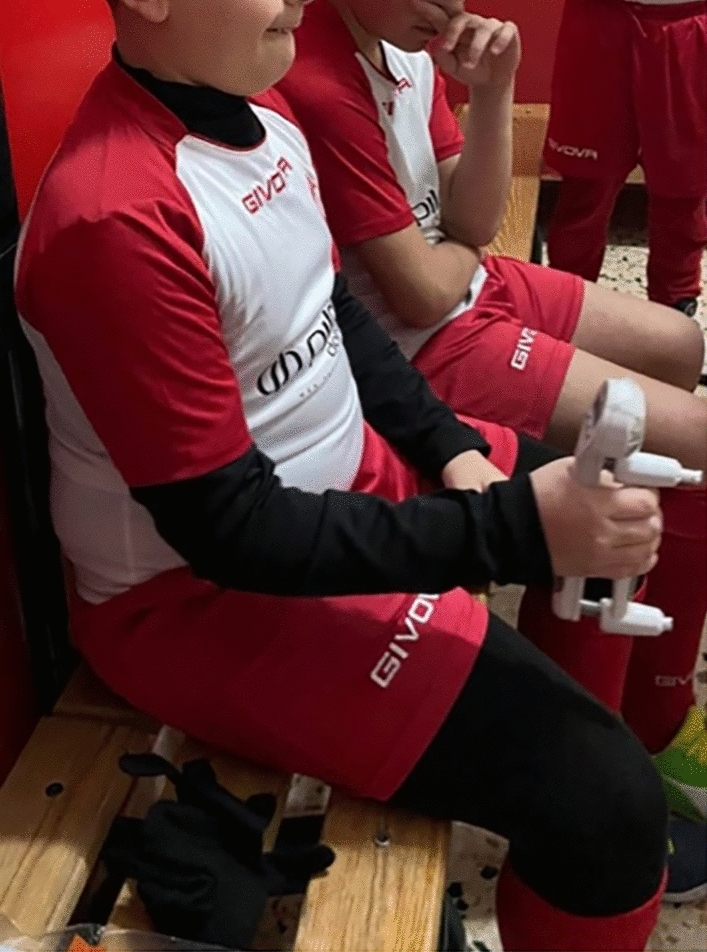
Figure represents the photos of one participant during the handgrip performance.

HGT is a test to assess the maximum strength of the upper limbs (kg). The instrument consists of a resistance (spring) and a handle. The resistance is adjusted according to the age of the participants. Specifically, each participant had to exert sufficient strength to create displacement but not cause contact between the parts of the instrument. Hence, the use of 20 kg spring was adequate for all participants. Each participant was asked to grip and squeeze the dynamometer expressing the maximum strength. The test was carried out in a sitting position, with the back resting on the chair and with a 90° angle between the arm and forearm^28–30^. The dynamometer used was the Kern Map model 80K1—Kern®, Kern & Sohn GmbH, Balingen, Germany. The test was repeated three times for both the dominant (d) and non-dominant (nd) limbs, and the best of the three trials for both the d and nd limbs was considered for statistical analysis^31–33^ (Fig. 1).

#### Coin rotation task (CRT)

**Figure 2:**
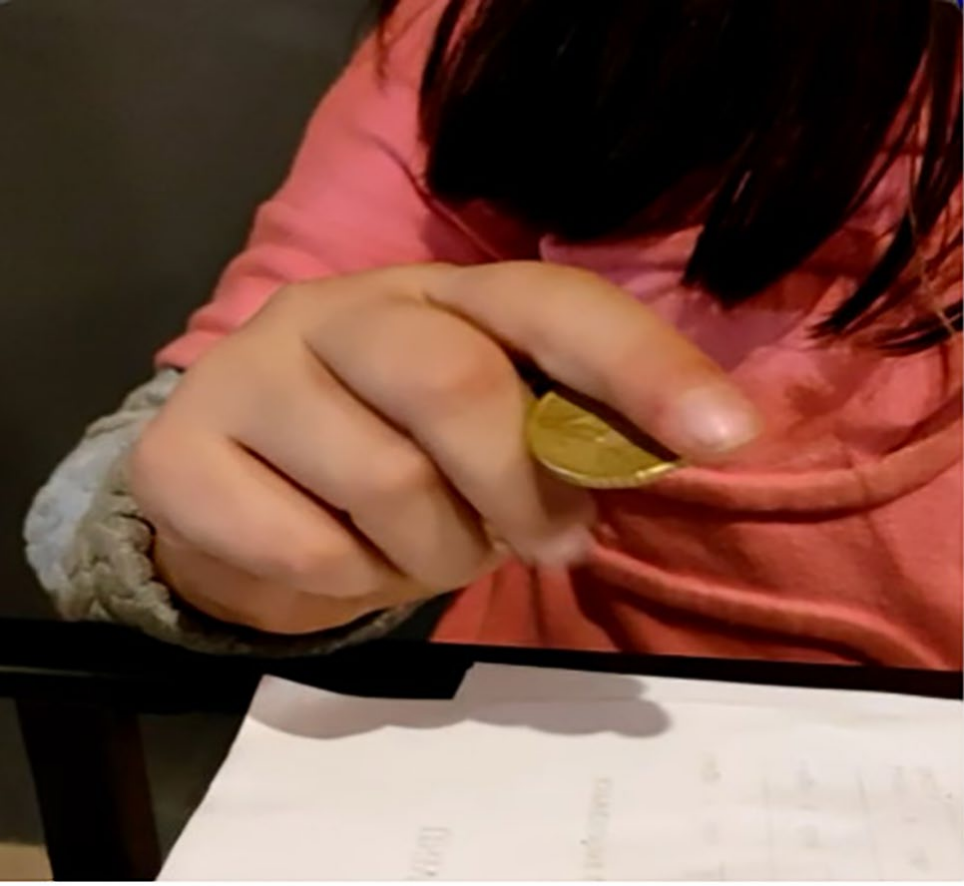
Figure represents the photos of one participant during the coin rotation task performance.

CRT is a test for the assessment of manual dexterity. Participants were asked to rotate a coin with a standardized diameter and weight (Nordic Gold (CuAl5Zn5Sn1), diameter of 24.25 mm, thickness 2.38 mm, weight 7.80 g) between the first three fingers of the hand. The test was performed in a sitting position, the non-working hand resting (palm down) on a table placed in front of the participants. The number of rotations was the score of this task^23^. The test was repeated three times for both the d and nd limbs, and the average of the three measurements for the d and nd limbs was considered for statistical analysis (Fig. 2).

#### Grooved pegboard test (GPT)

**Figure 3:**
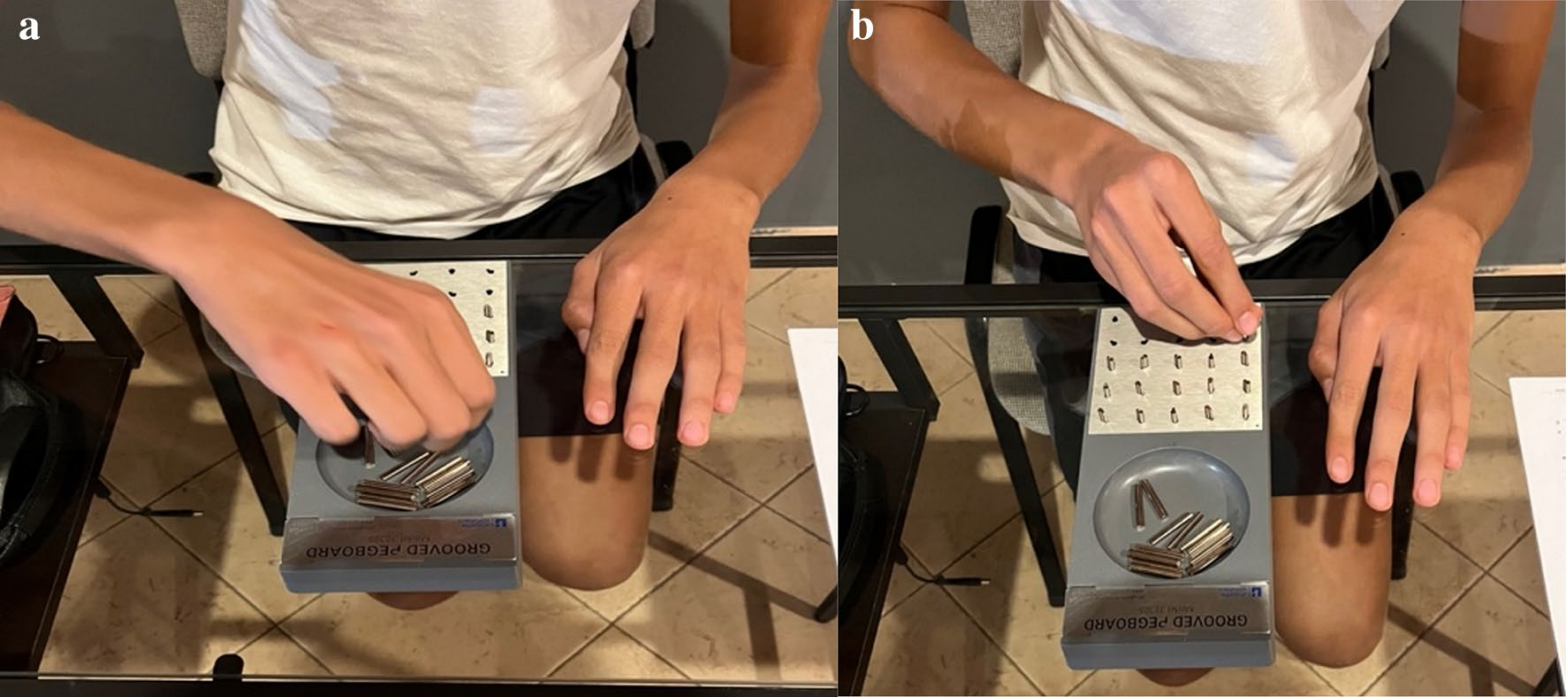
Figure represents the photos of the grooved pegboard test performance of one participant in the peg grip phase (**A**) and peg insertion phase (**B**).

GPT is a standardized test for the assessment of manual dexterity. Participants were asked to insert 25 pegs into a board with 25 holes in the shortest possible time, taking one peg at a time with only the limb we were testing. The test was repeated twice for both limbs and the sum of the time score (s) of the two tests for both limbs was considered for statistical analysis. The test was performed in a sitting position with the pegboard resting on a table placed in front of the participants as well as the non-working hand (palm down on the table). The instrument used was the Grooved Pegboard Model 32,025^1^ (Fig. 3).

### Statistical analysis

The Shapiro–Wilk test was used to test the normal distribution of the data in the three groups. The difference between the groups for each dependent variable (both *d* and *nd* performance of GPT, CRT, and HGT) was evaluated with a multivariate analysis of covariance (MANCOVA) with “age” as the covariate and “groups” as the factor. Dwass–Steel–Critchlow–Fligner pairwise comparison was used for variables whose data did not have a normal distribution. Power analysis for MANCOVA was performed using “G*Power 3.1” software, and the a priori sample size was calculated setting error probability α at 0.05, power β at 0.8, and effect size f^2^ (V) at 0.25.

The test–retest reliability of the CRT scores was assessed through the intra-class correlation (ICC_2,1_) coefficient (two-way absolute agreement) with a 95% confidence interval^23^. ICC > 0.90 indicates excellent reliability, ICC 0.75–0.90 indicates good reliability, ICC 0.50–0.75 indicates moderate reliability, and ICC < 0.50 indicates poor reliability. The SEM, which indicates random error in a measure on repeated assessments, was calculated as follows SEM: SD_pooled_ $\sqrt{1}-ICC$^34^. Construct validity was determined by the association between variables evaluated with Spearman’s Rho.

Statistical analysis was performed using “Jamovi” software version “2.3.21.0”.

### Ethics

The study design meets the purposes of the Declaration of Helsinki, and the Ethics Committee Commission of the University of Novi Sad Faculty of Sport and Physical Education approved the study (Approval number: 49-03-02/2023-01 Novi Sad, Serbia). Parents/guardians were informed about the study protocol and signed the informed consent for study participation. Informed consent was also obtained from all participants.

## Results

**Figure 4:**
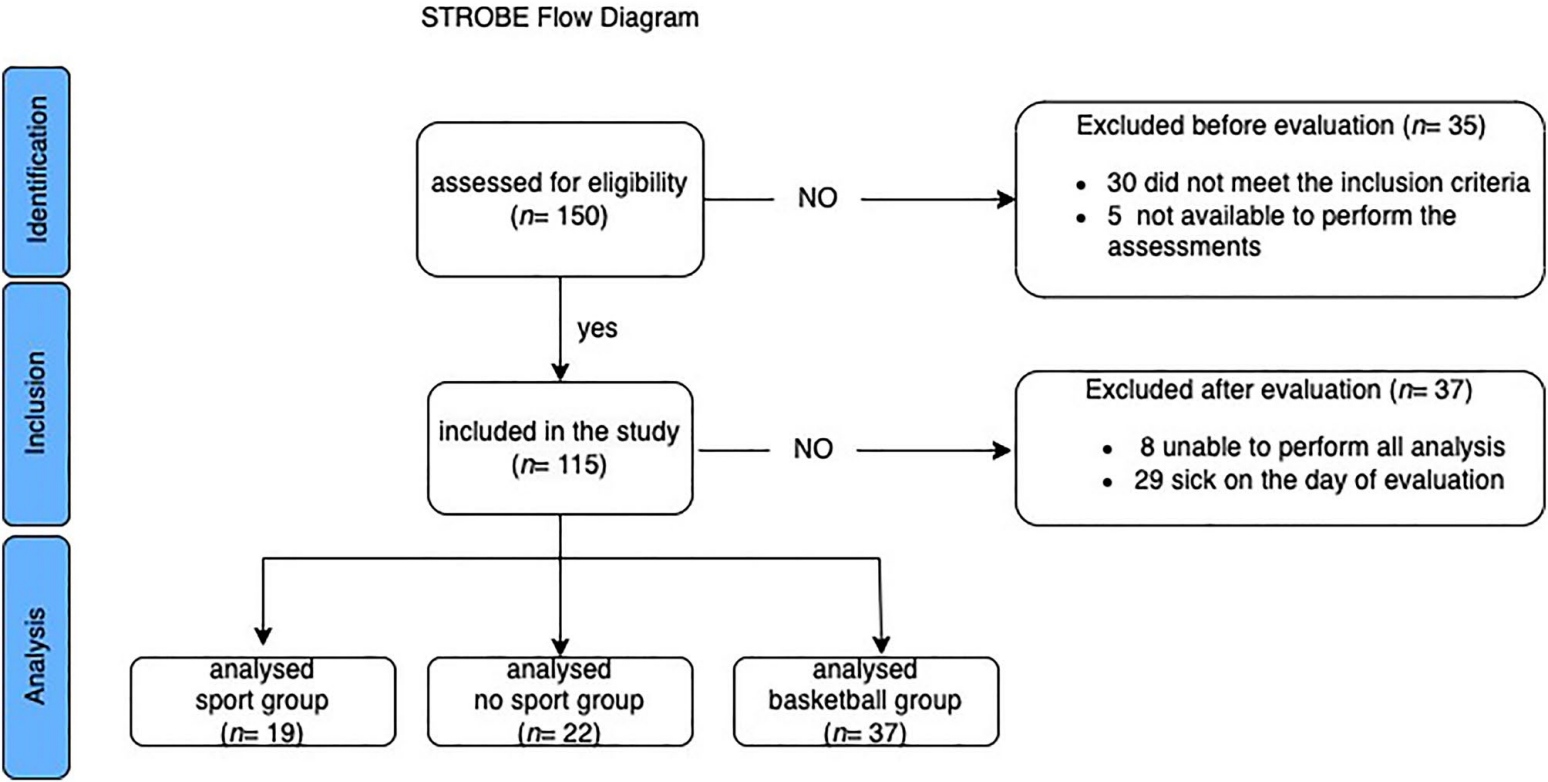
STROBE flow diagram of the participants’ recruitment process.

78 children (age: 12.45 ± 1.88 years) met the inclusion criteria and were included in our study. From the responses to the question “What kind of sports do you practice? And “for how long?” of the administered questionnaire, the participants were categorized into the groups as follows: n = 37 for the BG, n = 19 for the SG, and n = 22 for the nSG. The flow diagram describes the recruitment process (Fig. 4).

### Test–retest reliability and correlation analysis between dependent variables

Test–retest reliability of the CRT scores was moderate in the BG (ICC_2,1_ 0.63–0.61; CI 95% 0.44–0.78, 0.39–0.77), nSG (ICC_2,1_ 0.54–0.63; CI 95% 0.29–0.75, 0.41–0.81) and in the SG (ICC_2,1_ 0.60–0.57; CI 95% 0.34–0.80, 0.31–0.79). The SEM values were: in the BG 1.71–1.63, in the nSG 2.02–1.73, and in SG 1.47–1.67 (Table 2).

**Table 2:** Test–retest reliability of dominant and non-dominant coin rotation task (CRT) in basketball group, non-sport group, and sport group.

Group	Analysis	Mean SD			Range			ICC (CI _95%_)	SEM
		First assessment	Second assessment	Third assessment	First assessment	Second assessment	Third assessment		
Basketball players	CRT_dominant_	14.5 ± 2.90	15.1 ± 2.66	16.1 ± 2.88	6–19	10–22	10–23	0.63 (0.44–0.78)	1.71
	CRT_non-dominant_	13.1 ± 2.44	13.9 ± 2.70	14.8 ± 2.71	6–18	9–20	11–23	0.61 (0.39–0.77)	1.63
Non-sportsmen	CRT_dominant_	13.7 ± 3.57	15.0 ± 2.66	15.0 ± 2.70	6–20	10–19	11–20	0.54 (0.29–0.75)	2.02
	CRT_non-dominant_	12.8 ± 2.64	13.2 ± 3.13	13.5 ± 2.76	7–18	8–19	9–19	0.63 (0.41–0.81)	1.73
Sportsmen	CRT_dominant_	14.3 ± 2.16	14.7 ± 2.62	14.4 ± 2.19	10–19	10–19	11–18	0.60 (0.34–0.80)	1.47
	CRT_non-dominant_	12.8 ± 2.72	12.6 ± 2.39	13.2 ± 2.54	6–18	9–19	10–18	0.57 (0.31–0.79)	1.67

**Figure 5:**
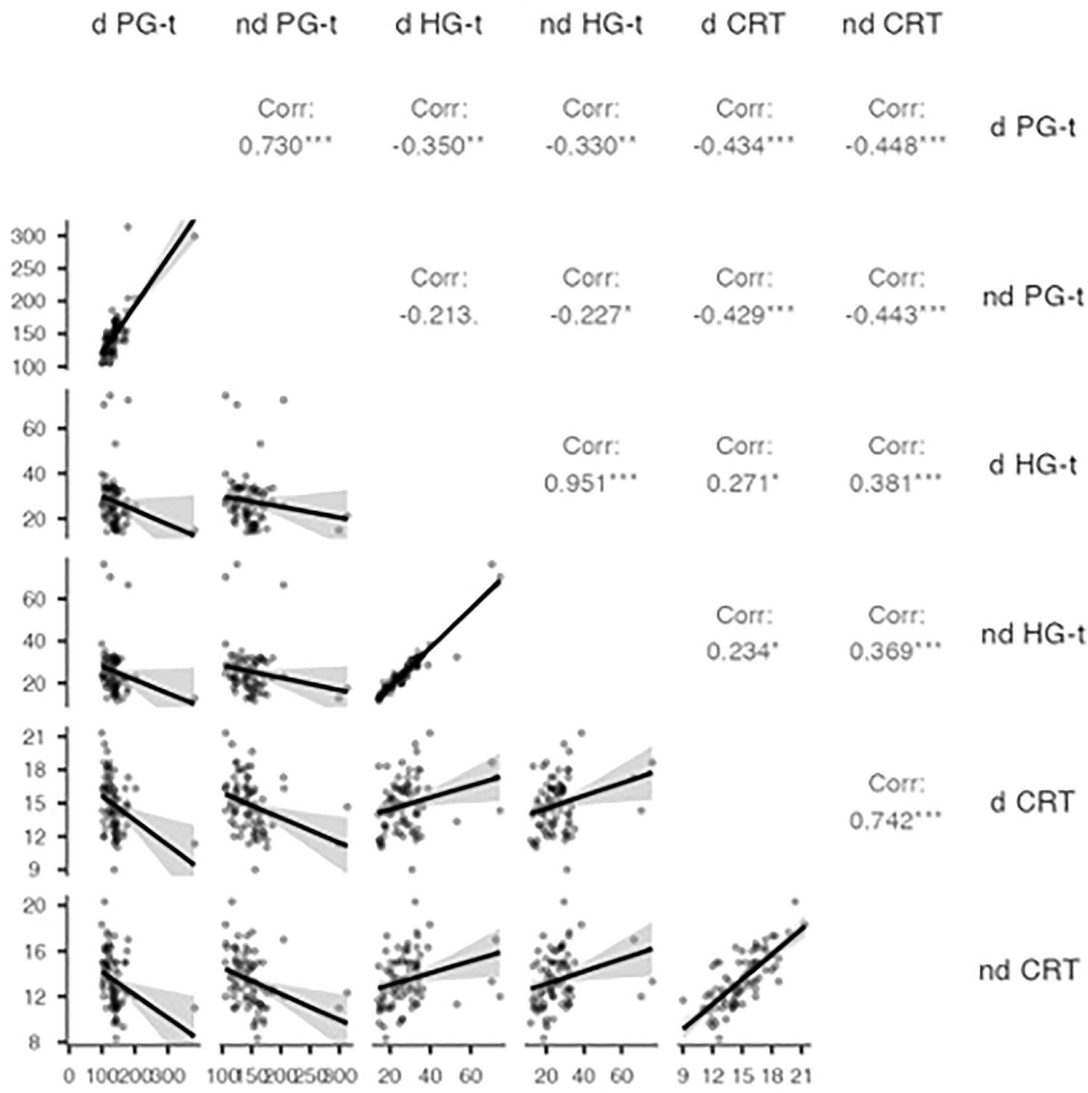
Correlation analysis between variables scores in the entire sample. Level of significance: *p < 0.05, **p < 0.01, ***p < 0.001.

The results of correlation analysis showed a significant low to moderate correlation^35^ between the *d* CRT and *d* HGT (rho = 0.3; p = 0.02; CI_95%_ 0.04–0.46) and between *nd* CRT and *nd* HGT (rho = 0.4; p < 0.001; CI_95%_ 0.05–0.46); the correlation between CRT and GPT was significant moderate between *d* CRT and *d* GPT (rho = − 0.4; p < 0.001; CI_95%_ − 0.51 to − 0.12) and *nd* CRT and *nd* GPT (rho = − 0.4; p < 0.001; CI_95%_ − 0.52 to − 0.12) (Fig. 5).

### Multivariate analysis of covariance

The overall results of the MANCOVA analysis, performed considering as variables the results of GPT, CRT, and HGT for both dominant and non-dominant limbs and the age as a covariate, showed significant group differences (Wilks λ = 0.65, *F*_*2,75*_, p = 0.002), univariate analysis results are shown in Table 3A. MANCOVA analysis results with significant covariate for age for CRT and HGT but not for GPT (Table 3B).

**Table 3:** MANCOVA analysis for the factor “groups” (A) and for the covariate “age” (B).

(A) Dependent variables	Factor “groups”			p-value
	SG (mean ± SD)	BG (mean ± SD)	nSG (mean ± SD)	
d Grooved Pegboard test	155 ± 58.9	123 ± 14.6	140 ± 24	0.00**
nd Grooved Pegboard test	155 ± 39.8	136 ± 20.7	159 ± 41.6	0.02*
d Handgrip test	21.5 ± 6.12	32.4 ± 11.7	25.1 ± 12.2	< 0.001***
nd Handgrip test	20.1 ± 5.51	29.8 ± 11.6	23.4 ± 11.4	0.00**
d Coin rotation task	14.5 ± 1.99	15.2 ± 2.49	14.6 ± 2.52	0.40
nd Coin rotation task	12.8 ± 2.15	13.9 ± 2.31	13.2 ± 2.47	0.18

(B) Covariate	Dependent variables	p-value		
Age	d Grooved Pegboard test	0.17		
	nd Grooved Pegboard test	0.19		
	d Handgrip test	0.00**		
	nd Handgrip test	< 0.001***		
	d Coin rotation task	0.51		
	nd Coin rotation task	0.01*		

*d* dominant limb, *nd* non-dominant limb, *SG* sports group, *BG* basketball group, *nSG* non-sports group, *SD* standard deviation; significances for MANCOVA: *p < 0.05, **p < 0.01, ***p < 0.001.

### Dwass–Steel–Critchlow–Fligner pairwise comparison

**Figure 6:**
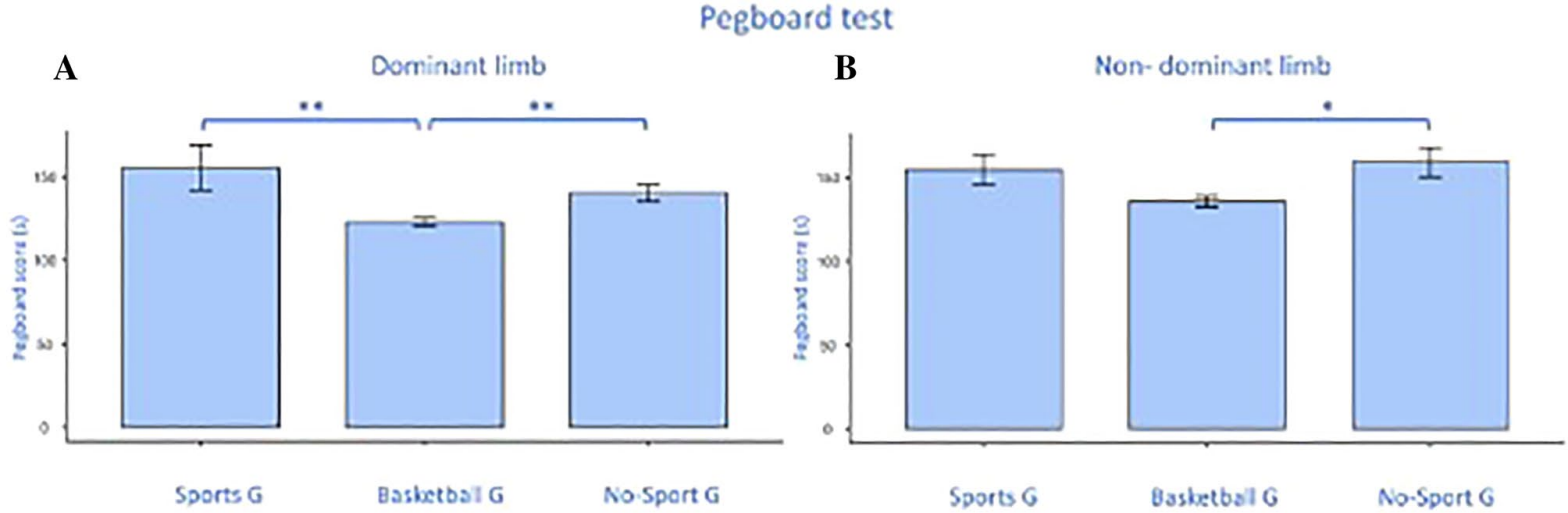
Pairwise comparison between groups for the grooved pegboard tests, for dominant (**A**) and non-dominant (**B**) limb scoring. Significances: *p < 0.05, **p < 0.01.

**Figure 7:**
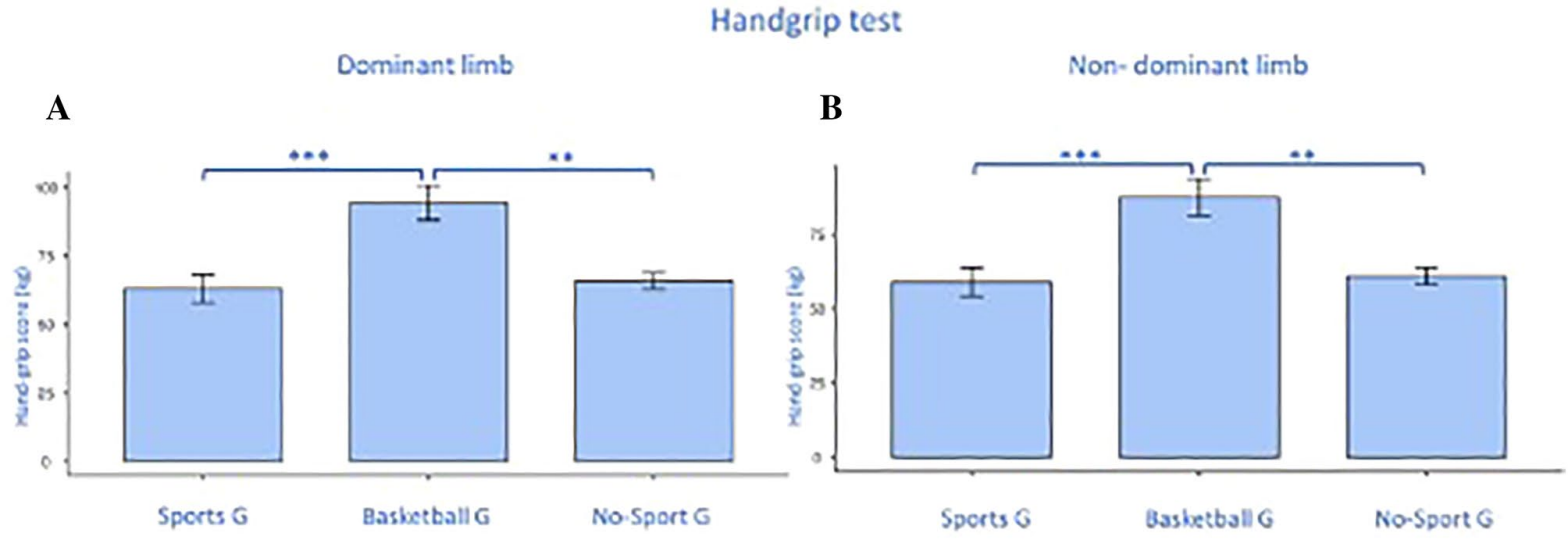
Pairwise comparison between groups for the handgrip tests, for dominant (**A**) and non-dominant (**B**) limb scoring. Significances: **p < 0.01, ***p < 0.001.

We performed the Dwass–Steel–Critchlow–Fligner pairwise comparison since the HGT and GPT scores, which had a significant univariate analysis for both limbs, had p < 0.001 at the Shapiro–Wilk test. The BG scored significantly higher than SG (p = 0.00) and nSG (p = 0.01) in dominant GPT but no differences were found between SG and nSG (Fig. 6A). As for the non-dominant limb for the GPT, a significant difference was found between BG and nSG (p = 0.02), (Fig. 6B). Both in d (Fig. 7A) and nd (Fig. 7B) limbs the HGT performance showed a significant difference between BG and SG (d: p < 0.001; nd: p < 0.001) and between BG and nSG (d: p = 0.00; nd: p = 0.00) with the higher scores for the BG (Fig. 7A,B).

## Discussion

The main purpose of this study was to evaluate whether young basketball players have better manual dexterity performance than sportsmen and no-sportsmen peers in three manual dexterity tests. Another purpose of the study was to test whether CRT could be a reliable, easy, and effective method to test the manual dexterity in young basketball players.

Our results showed that the sport group (SG), who did not perform basketball, did not differ from their non-sports peers (nSG) in the grooved pegboard test (p > 0.05), handgrip test (p > 0.05), and coin rotation task (p > 0.05). Our findings are in agreement with the results by Kuloor who found no significant difference between sportsmen and non-sportsmen people in hand–eye coordination assessed with the Minnesota Manual Dexterity Test (MMDT)^10^.

In our study basketball players (BG) differed significantly in HGT (p < 0.001) and GPT (p < 0.05) from nSG indicating that the type of sport could influence manual dexterity performance in young people. In fact, basketball stimulates manual dexterity more than other sports because it is characterized by movements such as dribbling, passing, catching, throwing, shooting, and movements in dynamic and changing environments that occur due to the control of the ball which occurs with the hands and cannot be found in other sports^13–15^. These movements cause adaptations of the areas of the central nervous system devoted to bimanual and eye-hand coordination^13,16^. Indeed, BG showed better performances than those who practiced other sports in the dominant GPT and in the dominant and non-dominant HGT. Furthermore, BG performed the best absolute value averaged score between the three groups even in the CRT for both dominant (SG: 14.5 ± 1.99; BG: 15.2 ± 2.49; nSG: 14.6 ± 2.52) and non-dominant (SG: 12.8 ± 2.15; BG: 13.9 ± 2.31; nSG: 13.2 ± 2.47) hand.

As regards the correlations between variables, we found a moderate correlation between CRT and GPT both for the dominant (p < 0.001) and non-dominant limbs (p < 0.001). These results are in agreement with Meimandi et al. who found the same correlation between CRT and the Purdue Pegboard Test for both hands in a sample of children with specific learning disorders^23^.

An experimental study by Mendoza et al. examined the convergent and discriminant validity of CRT compared to other standardized motor tasks aimed at measuring manual dexterity. The authors found no correlation between CRT and hand grip strength performances^24^. In contrast, our results showed a significant correlation between dominant CRT and HGT (p < 0.05) and between non-dominant CRT and non-dominant HGT (p < 0.001). This could mean that isometric strength of upper limbs may influence manual dexterity performance in young basketball athletes, but to confirm this further studies on the effect of isometric training on manual dexterity would be needed. However, the age of the population included in the study by Mendoza et al. was older than that of our sample.

Results of the present study on the reliability of the CRT showed a moderate test–retest reliability in young basketball players. The SEM values obtained for CRT were acceptable, suggesting that this test has sufficient accuracy and acceptable absolute reliability.

## Conclusions

To the best of our knowledge, this is the first study that investigated manual dexterity in young basketball players by comparing their performances with sportsmen and non-sportsmen peers. The basketball group showed better values in all manual dexterity tests with statistical significance compared with peers playing other sports and peers who did not play sports.

In conclusion, we can state that playing basketball can positively influence manual dexterity development in the young population. However, more studies are needed to investigate the influence of other sports and to include participants with a smaller age range. In addition, our was the first study using CRT as a reliable and simple test for measuring manual dexterity in basketball players. However, very few psychometric data exist regarding this task in children who play sports.

### Practical and future implications

Eye-hand coordination, bimanual and visuomotor coordination, and manual dexterity are the main skills required in basketball players^13–15^. These are fundamental to achieve a good level of performances in typical gestures of the sport such as dribbling, passing, catching, throwing, shooting, and movements in dynamic and changing environments^13–15^.

Monitoring manual dexterity could be useful to identify any deficiencies in the development of sport-specific skills, such as lack of manual dexterity. As our study showed, this could be done with a reliable, easier, cheaper, and less time-consuming tool.

Furthermore, it might be useful to know that a young person who has been playing basketball for at least two years can have higher scores than inactive peers or than those who play other sports. Hence, the practice of a sport can have a specific effect on the development of manual dexterity, and this aspect could be useful to parents for choosing a sport for their children. For example, children with impaired manual dexterity development have proven benefits in practicing physical therapy with movements such as catching, throwing, and shooting^11,12,23^. In this way, playing basketball could contribute to improve manual dexterity in an inclusive and non-experimental context.

### Strengths and limitations

This study has some limitations. First, the three groups do not have the same number of participants due to those excluded because they did not meet the inclusion criteria; the age range was wide to control for the possible influence of hormonal changes during the puberty period (especially for female participants); the tests were administered at different times of the day and this could affect manual dexterity performance. However, to the best of our knowledge, no previous studies examined CRT reliability in sportsmen, particularly in basketball players.

## Data Availability

Anonymous data is available upon request (Dr. Alessandra Amato; alessandra.amato02@unipa.it).
